# METTL3 recruiting M2-type immunosuppressed macrophages by targeting m6A-SNAIL-CXCL2 axis to promote colorectal cancer pulmonary metastasis

**DOI:** 10.1186/s13046-024-03035-6

**Published:** 2024-04-11

**Authors:** Peng Ouyang, Kang Li, Wei Xu, Caiyun Chen, Yangdong Shi, Yao Tian, Jin Gong, Zhen Bao

**Affiliations:** Department of General Surgery, The First Affiliated Hospital of Jinan University, Guangzhou, 510632 Guangdong China

**Keywords:** METTL3, N6-Methyladenosine, Epithelial–mesenchymal transition, Colorectal cancer pulmonary metastases, M2-type macrophages

## Abstract

**Background:**

The regulatory role of N6-methyladenosine (m6A) modification in the onset and progression of cancer has garnered increasing attention in recent years. However, the specific role of m6A modification in pulmonary metastasis of colorectal cancer remains unclear.

**Methods:**

This study identified differential m6A gene expression between  primary colorectal cancer and its pulmonary metastases using transcriptome sequencing and immunohistochemistry. We investigated the biological function of METTL3 gene both in vitro and in vivo using assays such as CCK-8, colony formation, wound healing, EDU, transwell, and apoptosis, along with a BALB/c nude mouse model. The regulatory mechanisms of METTL3 in colorectal cancer pulmonary metastasis were studied using methods like methylated RNA immunoprecipitation quantitative reverse transcription PCR, RNA stability analysis, luciferase reporter gene assay, Enzyme-Linked Immunosorbent Assay, and quantitative reverse transcription PCR.

**Results:**

The study revealed high expression of METTL3 and YTHDF1 in the tumors of patients with pulmonary metastasis of colorectal cancer. METTL3 promotes epithelial-mesenchymal transition in colorectal cancer by m6A modification of SNAIL mRNA, where SNAIL enhances the secretion of CXCL2 through the NF-κB pathway. Additionally, colorectal cancer cells expressing METTL3 recruit M2-type macrophages by secreting CXCL2.

**Conclusion:**

METTL3 facilitates pulmonary metastasis of colorectal cancer by targeting the m6A-Snail-CXCL2 axis to recruit M2-type immunosuppressive macrophages. This finding offers new research directions and potential therapeutic targets for colorectal cancer treatment.

In patients with colorectal cancer (CRC), cancer metastasis is the primary cause of cancer-related deaths, with the liver and lungs being the most common target organs for metastasis [1, 2]. While the majority of research has traditionally focused on liver metastases, colorectal cancer pulmonary metastasis (CRPM) has received relatively less attention. This gap in research is particularly significant given that the lungs are the second most common site of metastasis for CRC. Epidemiological studies estimate that pulmonary metastases occur in approximately 10–18% of rectal cancer patients and 5–6% of colon cancer patients [3]. The mechanisms of CRPM are multifaceted, encompassing intracellular factors such as genetic and epigenetic abnormalities, tumor cell heterogeneity, and the process of epithelial-mesenchymal transition (EMT). Furthermore, the influence of the tumor microenvironment cannot be underestimated [4]. Given its significance in cancer progression and treatment, in-depth research on CRPM is imperative. This not only contributes to a better understanding of the metastatic mechanisms in CRC but is also crucial for the development of novel therapeutic strategies.

In the formation process of CRPM, the EMT plays a crucial role [3]. EMT is a critical mechanism in the process of tumor initiation and development, and its activation is typically regulated by transcription factors that induce EMT. These factors include Snail family transcriptional repressor 1 (SNAIL) protein, Zinc finger E-box-binding homeobox 1 (ZEB) protein, and Twist family bHLH transcription factor 1 (TWIST1) protein [5–8]. This activation process leads to a series of changes in cells, including the loss of cell apical-basal polarity, disruption of cell–cell junctions, degradation of the basement membrane, and remodeling of the extracellular matrix (ECM) [9, 10]. These changes transform epithelial cells from their original cobblestone-like appearance into spindle-shaped mesenchymal cells [11]. At the molecular level, the characteristics of EMT include a decrease in epithelial markers such as E-cadherin and an increase in mesenchymal markers such as vimentin, N-cadherin, and fibronectin [12]. Clinically, these changes enhance the mobility and invasive capacity of tumor cells, thereby promoting the distant metastasis of colorectal cancer cells [13]. Therefore, in-depth research into the EMT process is crucial for understanding the progression mechanisms of colorectal cancer and developing effective personalized treatment strategies.

Epigenetic abnormalities play a crucial role in the occurrence of CRPM [3]. Indeed, N6-methyladenosine (m6A) modification is one of the most common forms of epigenetic modifications found in messenger RNA (mRNA) [14, 15]. Recent research has indicated that m6A plays a crucial role in various aspects of mRNA, including splicing, nuclear export, translation, and stability. Its significance extends to the development of many human diseases, including cancer [16, 17]. This modification is controlled by a group of specialized enzymes, including "writers" that add methyl groups and "erasers" that remove methyl groups [18]. In this context, the 'writers' complex comprises enzymes such as methyltransferase-like 3 (METTL3) and methyltransferase-like 14 (METTL14), which are responsible for transferring methyl groups onto RNA [19–22]. The demethylation of m6A is facilitated by enzymes such as obesity-related protein (FTO) and alkB homologue 5 (ALKBH5) [23, 24]. These modifications are recognized and interpreted by a group of proteins known as 'readers,' which include YTH domain family proteins and insulin-like growth factor 2 mRNA-binding proteins (IGF2BP) [25, 26]. These intricate interactions influence the behavior and function of RNA, thereby playing crucial roles in the development of CRPM.

During the development of tumors, m6A often exerts its influence on the expression of key genes associated with EMT, either directly or indirectly, leading to the promotion of tumor cell EMT and facilitating their progression and metastasis. [27–30]. The relationship between m6A and EMT in cancer cells has been extensively studied, but its role in CRPM remains unclear. In our study, we conducted transcriptome sequencing, quantitative PCR (qPCR), Western blotting (WB), and immunohistochemical (IHC) analysis on clinical specimens of colorectal primary tumors and their lung metastatic tumors. Additionally, we integrated data from The Cancer Genome Atlas (TCGA) database and observed an increased expression of m6A-related genes, such as METTL3 and YTHDF1. These observations prompted us to further explore the potential link between these changes and colorectal cancer lung metastasis. Through in vivo and in vitro experiments, we found that METTL3 could enhance the expression of SNAIL protein through m6A modification, thereby promoting EMT in tumor cells. Furthermore, we observed that tumor cells overexpressing SNAIL could promote the secretion of CXCL2 by activating the NF-κB pathway. Secreted CXCL2, upon binding to CXCR2 on the surface of M2-type macrophages, facilitated the infiltration of these immune cells into the tumor center. These findings provide a new perspective on understanding the molecular mechanisms underlying colorectal cancer lung metastasis and may have significant implications for the development of novel therapeutic strategies.

## Materials and methods

### Specimens, cell culture and treatment

The transcriptome sequencing data for CRC in this study were sourced from The Cancer Genome Atlas (TCGA) data portal (accessible at https://portal.gdc.cancer.gov/). Chromatin immunoprecipitation (ChIP) sequencing data were obtained from the Gene Expression Omnibus (GEO) database (see https://www.ncbi.nlm.nih.gov/gds/). In situ carcinoma and lung metastatic carcinoma tissue specimens from colorectal cancer lung metastasis patients were provided by the First Affiliated Hospital of Jinan University. Collection of all specimens received approval from the ethics committee of the First Affiliated Hospital of Jinan University, and written informed consent was obtained from the patients before collection.

CRC cell lines used in this study, including RKO and SW480, as well as the human monocytic cell line THP-1, were all obtained from the American Type Culture Collection (ATCC, Manassas, USA) (details available at https://www.atcc.org/). THP-1 cells were treated with 100 ng/ml phorbol 12-myristate 13-acetate (PMA, Sigma, Darmstadt, Germany) for 48 h to induce their differentiation into mature macrophages. Subsequently, the cells were incubated for over 48 h under conditions containing 20 ng/ml interleukin-4 (IL-4, Sigma, Darmstadt, Germany) and 20 ng/ml interleukin-13 (IL-13, Sigma, Darmstadt, Germany) to promote their polarization towards the M2 phenotype. All cell lines were cultured following the guidelines recommended by ATCC.

### Production of lentiviruses

The lentiviral transfection vectors used in this study were obtained from GenePharma (Shanghai, China). Experimental groups included sh-METTL3 (METTL3 knockdown group), sh-YTHDF1 (YTHDF1 knockdown group), sh-SNAIL (SNAIL knockdown group), and Ov-SNAIL (SNAIL overexpression group), along with their corresponding negative control group sh-NC. To establish stable colorectal cancer cell lines, cell selection was performed using 400 μg/ml of neomycin.

### Wound-healing assays

In this experiment, cells were initially seeded in 6-well plates. When the cells reached approximately 80% confluence, sterile 200 μl pipette tips were gently used to create scratches in the cell monolayer. Subsequently, these treated cells were incubated for an additional 48 h in serum-free culture medium. The healing process of cell injuries was observed using an inverted microscope produced by Olympus Corporation, Japan. Finally, quantitative analysis of the wound area was performed using ImageJ software to assess cell migration and healing ability.

### Transwell invasion assay

In our cell invasion experiments, we utilized Transwell chambers and Matrigel from Corning, USA. Initially, a layer of extracellular matrix gel was coated in the upper chamber of the Transwell, and medium containing 20% FBS was added to the lower chamber. Once the gel had completely solidified, we added 200 µL of medium containing 1 × 10^5 cells to the upper chamber. Subsequently, the cells were incubated at 37 °C with 5% CO2 for 48 h. After the incubation period, we fixed the cells in the upper chamber with 4% paraformaldehyde and then stained them with 1% crystal violet. Finally, we observed the cells that had invaded from the upper chamber to the lower chamber using an inverted microscope produced by Olympus Corporation, Japan, and conducted quantitative analysis using ImageJ software.

### Cell counting kit-8 (CCK-8) and EDU assay

In the CCK-8 experiment, cells were evenly distributed in a 96-well plate and cultured for 1, 2, 3, 4, and 5 days. Subsequently, 10 µL of CCK-8 solution was added to each well. To assess cell viability, the optical density (OD450) of each well was measured using a microplate reader from Thermo Fisher, USA.

For the EDU experiment, cell suspensions were evenly distributed in a 96-well plate to achieve a cell count of 1 × 10^4 cells per well. The culture dishes were then incubated overnight in a CO2 incubator to allow the cells to adhere to the surface. After a 2-h incubation with 10 µM EDU (Beyotime, Shanghai, China), the culture medium was removed, and 1 mL of fixation solution (Beyotime, Shanghai, China) was added to each well. The fixation solution was left at room temperature for 15 min and then removed. The cells were subsequently washed three times with 1 mL of washing solution. After removing the washing solution, 1 mL of Triton X-100 (Beyotime, Shanghai, China) was added to each well and incubated at room temperature for 10–15 min. Next, the Click reaction solution (Beyotime, Shanghai, China) was added and incubated in the dark at room temperature for 30 min. Finally, Hoechst 33,342 (Beyotime, Shanghai, China) was used for nuclear staining. Cell counting and photography were performed using a fluorescence inverted microscope, and quantitative analysis was conducted using ImageJ software.

### Clone formation assays

To collect cells during the logarithmic growth phase, a cell suspension was prepared using standard digestion and centrifugation methods. After cell counting, the cells were seeded into a 6-well plate at a concentration of 500 cells per well. Once the cells reached the 6th generation, culturing was stopped. The culture medium was discarded, and cells were washed twice with PBS solution. Subsequently, the cells were fixed in methanol for 15 min and stained with 1% crystal violet solution for 10 min. After capturing photographs with a camera, quantitative analysis was performed using ImageJ software.

### Apoptosis assays

In the apoptosis experiment, the detection of cell apoptosis was performed using the FITC Annexin V Cell Apoptosis Detection Kit (provided by BD Biosciences). The experimental steps were as follows: After 48 h of shRNA viral infection, the cells were seeded into 6 cm culture dishes. Cells were then cultured in medium containing 1% fetal bovine serum for 5 days. Following this, cells were digested using trypsin and mixed with the culture supernatant. After digestion was completed, cells were centrifuged, and the cell pellet was washed with PBS buffer to remove trypsin. Subsequently, the cells were resuspended and subjected to Annexin V/PI double staining. Finally, flow cytometry analysis of the samples was performed using the BD LSRII flow cytometer (BD Biosciences). Throughout the entire experiment, precautions should be taken to avoid exposure to light, and strict adherence to laboratory safety regulations is essential.

### M2-type macrophage migration assay

M2-type macrophage (1 × 10^5 cells per well) were plated in the upper compartment of a transwell with a pore size of 0.8 mm, in 100 mL of 1640 medium. The lower compartment was filled with 600 mL of conditioned medium collected from RKO or SW480 cells, excluding fetal bovine serum. Following a 4-h incubation period, the migrated cells in the lower chamber were quantified.

### RNA stability

Add Act-D to the cell culture medium at a concentration of 5 µg/mL when the cells have reached a confluence of 70–80%. Collect cell samples at different time points after Act-D treatment. Use TRIzol reagent (Invitrogen) to extract total RNA from the collected samples. Quantify the RNA and assess its purity using a NanoDrop spectrophotometer. Convert the extracted total RNA into cDNA using a reverse transcription reagent kit (Vazyme, Nanjing, China) following the manufacturer's instructions. Perform quantitative reverse transcription-polymerase chain reaction (qRT-PCR) analysis to detect the mRNA levels of specific genes (please specify the genes of interest). Normalize the data using GAPDH as the reference gene. Calculate the relative expression levels using the 2^-ΔΔCT method. Create line graphs illustrating the change in RNA levels over time to reflect RNA stability. Ensure that you follow the manufacturer's protocols for reagent usage and experimental procedures.

### Western blotting and immunohistochemistry

Cell and tissue samples were processed using RIPA buffer from Servicebio Technology, Wuhan, China. Protein extraction was carried out following the instructions provided in the radio-immunoprecipitation assay (RIPA) kit. Protein concentrations were quantified using the bicinchoninic acid (BCA) assay kit also provided by Servicebio Technology, Wuhan, China. The protein samples were subsequently separated by electrophoresis and transferred onto membranes. After washing the membranes three times with tris-buffered saline containing Tween (TBST), they were incubated with the primary antibody overnight at 4 °C. Following incubation, the membranes were washed again and incubated with the secondary antibody. Finally, protein bands were visualized using the enhanced chemiluminescence (ECL) assay kit provided by Servicebio Technology, Wuhan, China.

Immunohistochemistry and immunofluorescence as previously described [31]. In our study, immunohistochemistry was conducted using a range of primary antibodies with the following detailed information: 1. METTL3, Vimentin, E-Cadherin, YTHDF1, YTHDF2, SNAIL, and ARG1 antibodies were all obtained from Abcam (Cambridge, UK), each with the following product codes: METTL3 (ab195352), Vimentin (ab92547), E-Cadherin (ab40772), YTHDF1 (ab252346), SNAIL (ab180714), and CD163 (ab316218). 2. P65, Phospho-P65, GAPDH, and Tubulin antibodies were sourced from Proteintech Group (Chicago, USA) and were identified by the specific product codes: P65 (80,979–1-RR), Phospho-P65 (82,335–1-RR), GAPDH (10,494–1-AP), and Tubulin (11,224–1-AP). 3. Goat anti-rabbit IgG was purchased from Biosharp Life Sciences (Beijing, China) and had the product code BL052A. The quantification of all proteins and determination of average optical density were performed using Image J software.

### Luciferase reporter assays

The dual-luciferase reporter gene assay was conducted using the Dual-Luciferase® Reporter Gene Assay System provided by Promega Corporation. To standardize transfection efficiency, a co-transfection approach with the pRL-TK reporter gene (also supplied by Promega) was employed. Relative luciferase activity was calculated based on the activity of Renilla luciferase.

### mRNA-sequencing and methylated RNA immunoprecipitation quantitative reverse transcription polymerase chain reaction (MeRIP qRT-PCR)

Total RNA was extracted from colorectal cancer cell lines using the TRIzol reagent provided by Invitrogen Corporation (Carlsbad, CA, USA). Subsequently, mRNA sequencing and analysis were carried out by the HaploX Genomics Center in Shenzhen, China.

For RNA immunoprecipitation (MeRIP) analysis of m6A modification, we initially subjected the RNA to fragmentation using the riboMIP™ m6A Transcriptome Analysis Kit (Ribobio, Guangzhou, China) and then performed immunoprecipitation with m6A antibodies. The RNA isolated after immunoprecipitation was analyzed using qRT-PCR to investigate the m6A modification status of RNA.

### RNA Extraction and quantitative reverse transcription polymerase chain reaction (qRT-PCR)

Total RNA was extracted using TRIzol (Invitrogen, Carlsbad, USA), while cytoplasmic and nuclear RNA were extracted using cytoplasmic & nuclear RNA purification (Amyjet Scientific, Wuhan, China). qRT-PCR was performed following the protocol (Vazyme, Nanjing, China).

### ELISA

The cell culture medium was transferred to a sterile centrifuge tube and centrifuged at 1000 × g for 10 min at 4 °C. Aliquots of the supernatant were then dispensed into EP tubes for further use. The CXCL2 protein was detected using the manufacturer's protocols for the CXCL2 ELISA kit (Solarbio, Beijing, China).

### Animal studies

The experiments conducted in this study received ethical approval from the Ethics Committee of South China Agricultural University. Female nude mice aged 4 to 5 weeks were procured from the Animal Experiment Center of Southern Medical University for the experimental procedures. During the experimentation, a total of 10 nude mice were randomly divided into two groups, each consisting of 5 mice (n = 5). they were subcutaneous injected with 2 × 10^6 RKO/SW480 cells stably silencing METTL3 or control cells. Additionally, six nude mice were randomly assigned to two groups (n = 3), and they were intravenously injected with 2 × 10^6 RKO/SW480 cells stably silencing METTL3 or control cells. These nude mice were housed under standard laboratory conditions with access to ample food and water. Tumor size and volume were monitored weekly throughout the study. Before sample collection, humane euthanasia was administered to all mice using a 2% pentobarbital sodium solution at a dose of 150 mg/kg. After a 2-week interval, mice from the subcutaneous tumor group were euthanized due to tumor development. Similarly, mice from the intravenous tumor group were euthanized at the 4-week mark. The tumors were documented through photographic imaging and weighed. Subsequently, all collected tumor specimens were stored either in liquid nitrogen or fixed using a 4% formaldehyde solution for subsequent analysis.

### Statistical analysis

In this study, all statistical analyses were performed using SPSS 19.0 software and R language (version 4.2.1). Graphs and data visualization were carried out using GraphPad Prism 9.5 software. We defined a *p*-value of less than 0.05 as indicating statistically significant differences.

## Results

### METTL3 and YTHDF1 are highly expressed in CRPM

**Fig. 1:**
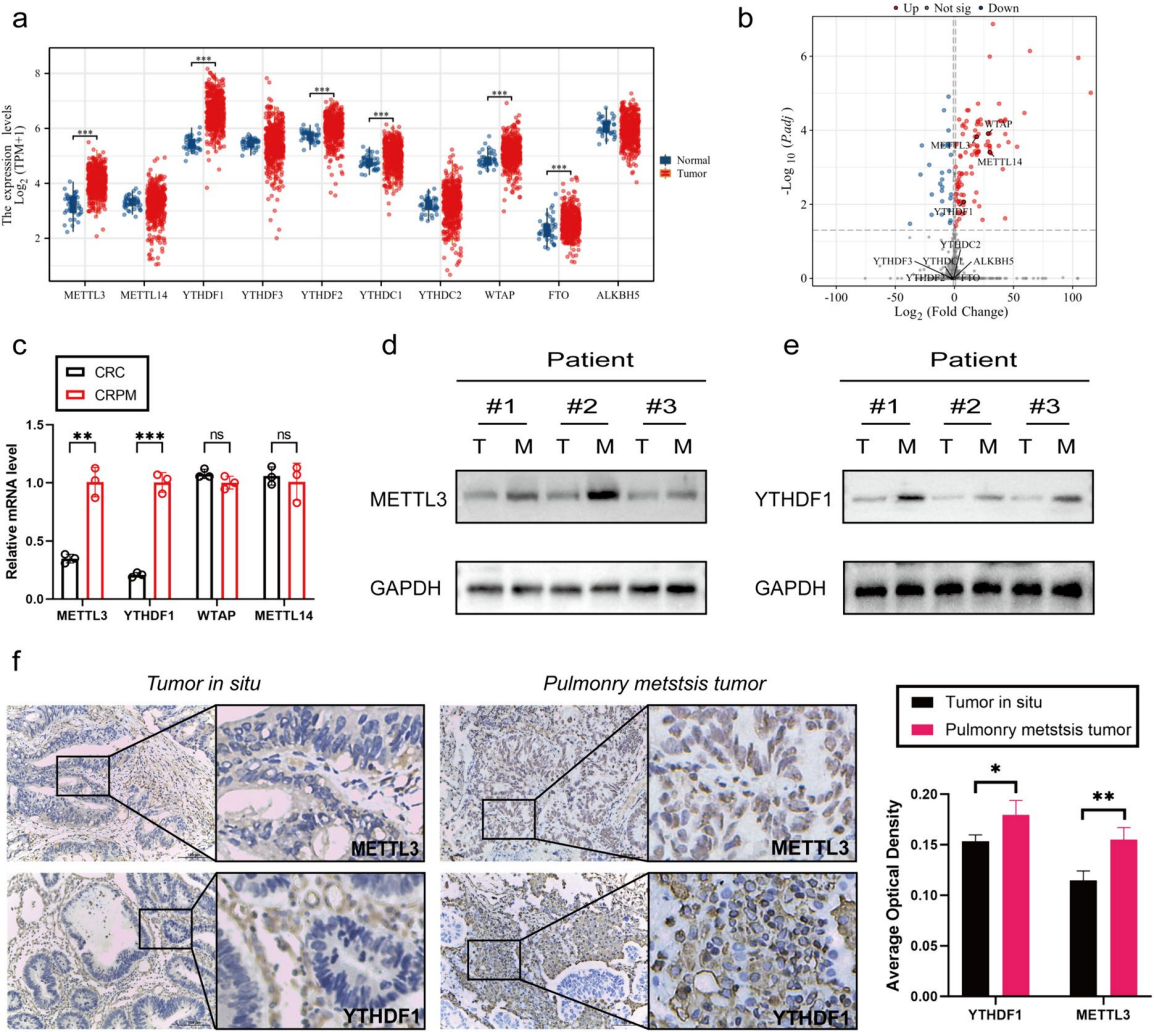
METTL3 and YTHDF1 are highly expressed in CRPM. **a** Expression profile of WERs in COAD based on TCGA database. **b** Volcano plot of differential gene sequencing WERs in colorectal cancer in situ and lung metastatic cancer. **c** qPCR Analysis of METTL3, WTAP, METTL14, and YTHDF1 Gene Expression in colorectal In Situ Carcinoma and Pulmonary Metastatic Carcinoma. **d-e** Western blotting analysis of METTL3 and YTHDF1 protein expression in colorectal in situ carcinoma and pulmonary metastatic carcinoma. T: colorectal In Situ Carcinoma. M: colorectal pulmonary metastatic carcinoma. **f** Immunohistochemical Analysis of METTL3 and YTHDF1 Protein Expression in colorectal In Situ Carcinoma and Pulmonary Metastatic Carcinoma. *P < 0.05, **P < 0.01, ***P < 0.001, ****P < 0.0001

To assess the expression profiles of m6A WERs (writers, erasers, and readers) in colon adenocarcinoma (COAD), this study analyzed data from The Cancer Genome Atlas (TCGA) database. The analysis results revealed that several m6A WERs exhibited abnormal expression in CRC (Fig. 1a). To further investigate the status of these differentially expressed genes in colorectal cancer in situ and lung metastatic cancer, we conducted sequencing on both types of cancer samples. The sequencing results indicated a significant increase in the expression of METTL3, wilms tumor 1 associated protein (WTAP), METTL14, and YTHDF1 in lung metastatic cancer. It's noteworthy that METTL14 did not show expression differences in the TCGA database (Fig. 1b).

To further validate these findings, we conducted qPCR analysis on colorectal cancer in situ and lung metastatic cancer samples. The qPCR results demonstrated that the expression of METTL3 and YTHDF1 was significantly higher in colorectal cancer lung metastases compared to in situ cancer, while WTAP expression showed no significant difference (Fig. 1c). Additionally, through WB and IHC experiments, we further confirmed the high expression of METTL3 and YTHDF1 in colorectal cancer lung metastases (Fig. 1d-f).

### Silencing the METTL3 gene inhibited the proliferation, invasion, and migration abilities of CRC cells while promoting apoptosis

**Fig. 2:**
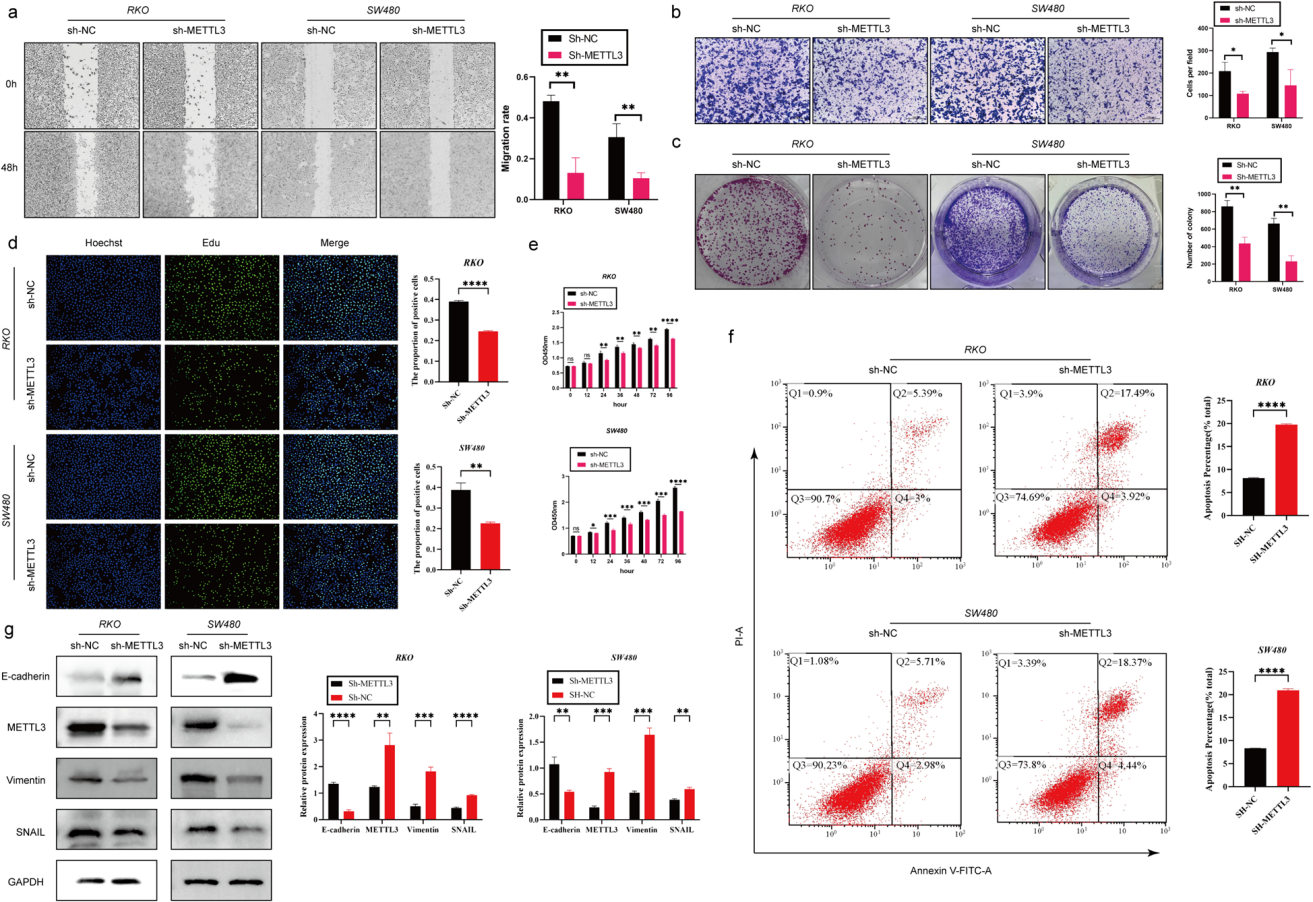
Silencing the METTL3 gene inhibited the proliferation, invasion, and migration abilities of CRC cells while promoting apoptosis. **a** Representative images and quantitative analysis of CRC cell migration based on wound healing assay.** b** Decreased expression of METTL3 restrained CRC cell invasion ability based on transwell assay. **c** The colony formation ability of CRC cells silencing METTL3 or not was measured by colony formation assay. **d** EDU assay in CRC cells was performed the to measure the proliferation level. **e** CCK-8 assay was applied to measure the proliferation level of CRC cells after transfected with sh-METTL3 or not. **f** Flow cytometry analysis the proportion of apoptosis. **g** Western blotting analysis was applied to measure the protein expression level of E-cadherin, METTL3, SNAIL and Vimentin. GAPDH was used as a loading control. *P < 0.05, **P < 0.01, ***P < 0.001, ****P < 0.0001

**Fig. 3:**
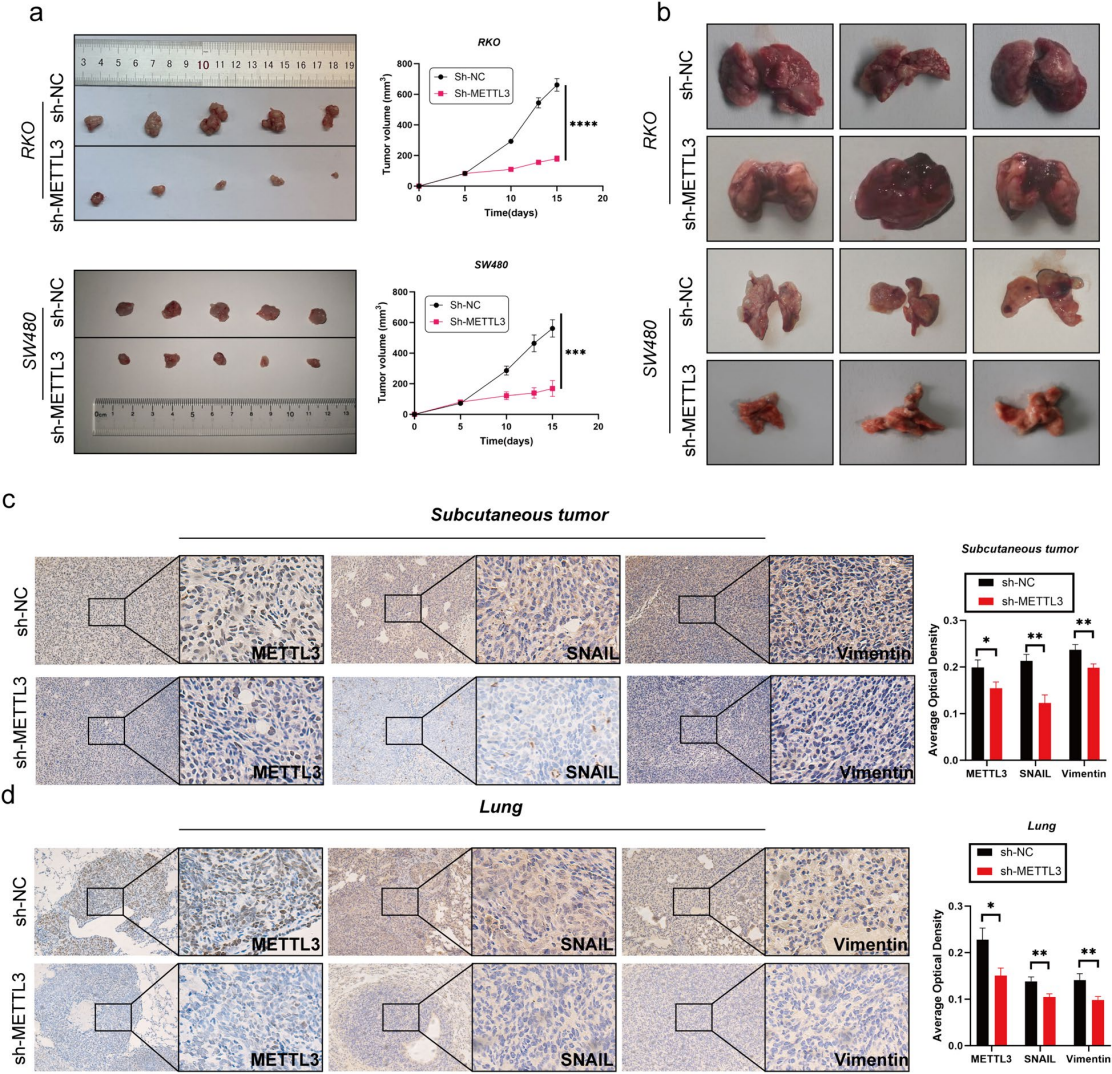
Decreased expression of METTL3 inhibits tumor growth in vivo. **a** Comparison of subcutaneous tumor size in nude mice after injecting RKO/SW480 CRC cells stably transfected with Sh-NC/Sh-METTL3. **b** The tail vein-lung metastasis model. Cells were injected into the tail vein to produce tumor cell lung metastasis. **c-d** IHC analysis of METTL3, SNAIL and Vimentin for tissues of subcutaneous tumor and lung metastasis tumor. *P < 0.05, **P < 0.01, ***P < 0.001, ****P < 0.0001

In the initial stages of this study, we successfully silenced the METTL3 gene in RKO and SW480 cell lines using lentiviral infection technology. To assess the silencing effect, we performed Western blot analysis (Fig. 2g). Subsequent cell proliferation experiments, including CCK-8, EDU staining, and plate colony assays, showed that after METTL3 knockdown, the OD450 values significantly decreased, the number of EDU-positive cells and cell colony formation were reduced (Fig. 2c-e). These results indicate that downregulation of METTL3 weakened the proliferation ability of RKO and SW480 cells. We further investigated the impact of METTL3 on apoptosis in CRC cells. Through Annexin V-APC/PI staining experiments, we observed a significant increase in cell apoptosis rate after METTL3 inhibition (Fig. 2f). Additionally, in vivo experiments using a nude mouse model showed that silencing METTL3 significantly reduced the size of subcutaneous tumors (Fig. 3l). In wound healing and Transwell experiments, we found that METTL3 knockdown in CRC cell lines resulted in weakened migration and invasion abilities (Fig. 2a-b). Another in vivo experiment also confirmed that inhibiting METTL3 significantly reduced the number of lung metastases in nude mice (Fig. 3b). To assess whether cells underwent EMT, we conducted Western blot analysis, which revealed that after METTL3 knockdown, the expression of VIM and Snail proteins significantly decreased, while E-cadherin protein expression significantly increased (Fig. 2g). Immunohistochemistry on tumor specimens from in vivo experiments also suggested that in the low-expression METTL3 group, both subcutaneous tumors and lung metastases showed significantly reduced expression of VIM and SNAIL proteins (Fig. 3c-d).

### METTL3 regulates tumor EMT process via m6A-Snail

**Fig. 4:**
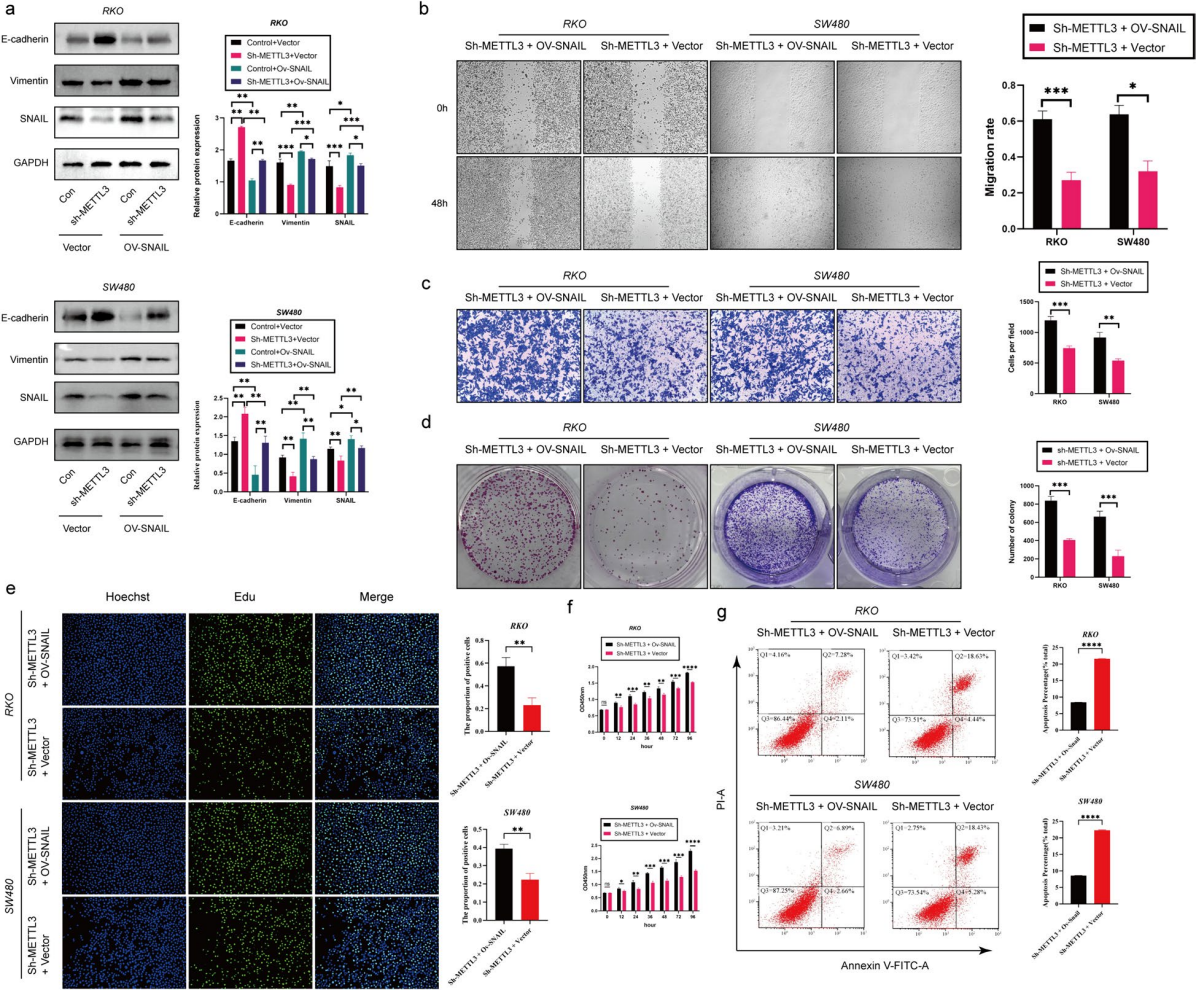
METTL3 regulates tumor process via Snail. **a** Western blotting analysis was applied to measure the protein expression level of E-cadherin, METTL3, SNAIL and Vimentin. GAPDH was used as a loading control. **b** Representative images and quantitative analysis of CRC cell migration based on wound healing assay. **c** Increased expression of SNAIL in sh-METTL3 CRC cell promoted CRC cell invasion ability based on transwell assay. **d** The colony formation ability of CRC cells overexpressing SNAIL or not was measured by colony formation assay in sh-METTL3 CRC cell. **e** EDU assay in CRC cells was performed the to measure the proliferation level. **f** CCK-8 assay was applied to measure the proliferation level of CRC cells after transfected with ov-SNAIL or not in sh-METTL3 CRC cell. **g** Flow cytometry analysis the proportion of apoptosis. *P < 0.05, **P < 0.01, ***P < 0.001, ****P < 0.0001

According to previous studies, SNAIL plays a crucial role in the tumor microenvironment [31]. Furthermore, existing research suggests a mutual regulatory relationship between METTL3 and Snail in various types of cancer [28]. Based on this background, our study aimed to investigate whether METTL3 in colorectal cancer (CRC) exhibits a similar regulatory relationship with Snail. Our hypothesis is that METTL3 controls epithelial-mesenchymal transition (EMT) in tumors through the m6A-Snail pathway. To validate this hypothesis, we conducted Snail overexpression experiments (ov-Snail) in sh-NC (control group) and sh-METTL3 (METTL3 silenced group) cells. Western blot analysis showed that silencing METTL3 attenuated Snail expression, and the EMT process could be reversed, while overexpressing Snail could restore EMT (Fig. 4a). This finding strongly supports the hypothesis that METTL3 regulates the EMT process through Snail. Additionally, we conducted SNAIL overexpression experiments in sh-METTL3 RKO and SW480 cell lines. The results showed that SNAIL overexpression reversed the inhibitory effect of silenced METTL3 on the proliferation, migration and invasion of CRC cells and its promotion of apoptosis (Fig. 4b-g).

**Fig. 5:**
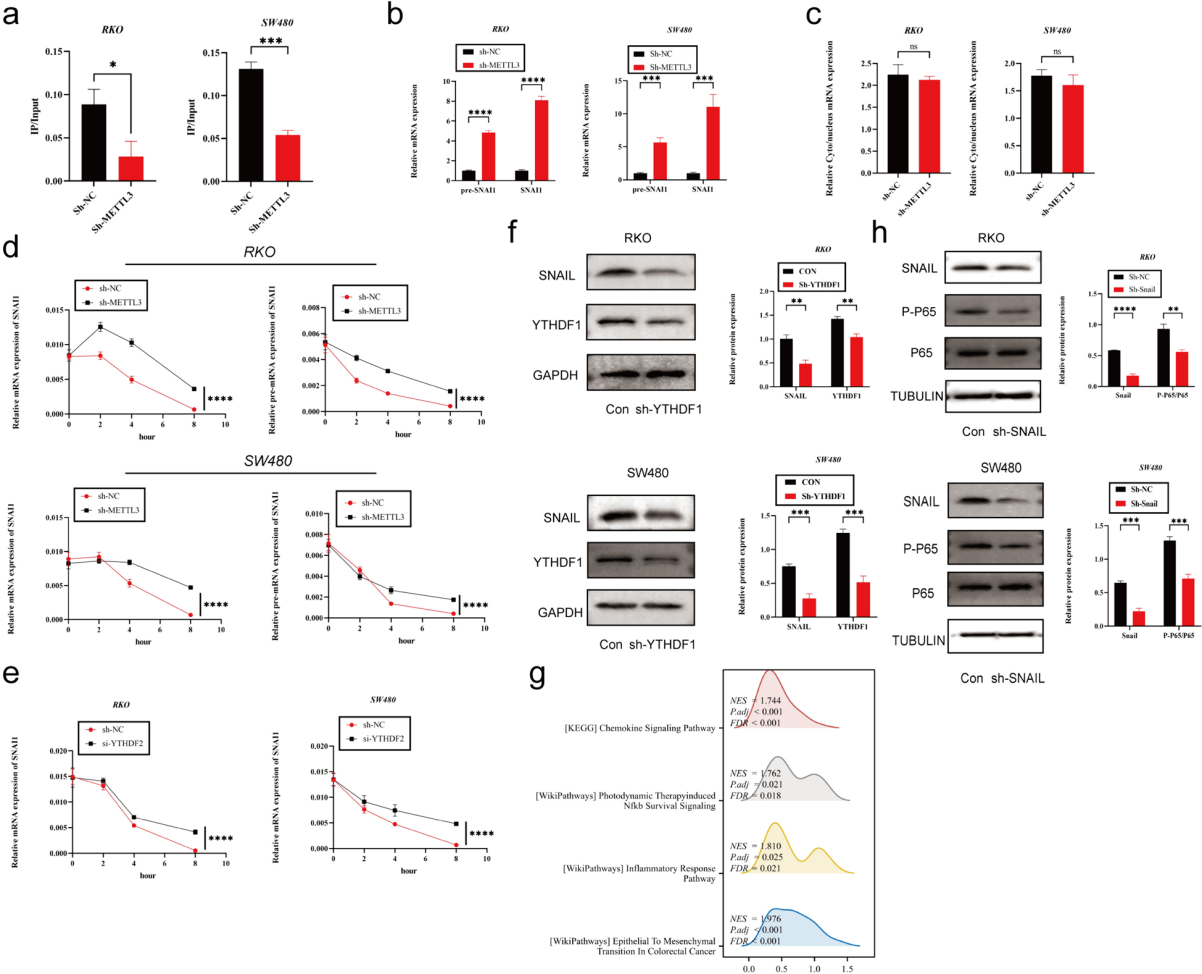
METTL3 regulates tumor EMT process via m6A-Snail. **a** The MeRIP qRT-PCR analysis of SNAIL mRNA in sh-NC and sh-METTL3 CRC cells. **b** Precursor and mature mRNA of SNAI1 in sh-NC and sh-METTL3 CRC cells. **c** The relative levels of the nuclear versus cytoplasmic SNAI1 mRNA in sh-NC and sh-METTL3 CRC cells. **d** sh-NC and sh-METTL3 CRC cells were pretreated with Act-D for 90 min, then precursor (right) or mature (left) SNAIL mRNA were analyzed at indicated times. **e** sh-NC and sh-YTHDF2 CRC cells were pretreated with Act-D for 90 min, then mature SNAI1 mRNA were analyzed at indicated times. **f** Western blotting analysis was applied to measure the protein expression level of SNAIL and YTHDF1. GAPDH was used as a loading control. **g** GSEA analysis of SNAIL high-expression and low-expression groups utilizing TCGA COAD data. **h** Western blotting analysis was applied to measure the protein expression level of SNAIL, P65 and P-P65. Tubulin was used as a loading control. *P < 0.05, **P < 0.01, ***P < 0.001, ****P < 0.0001

To further explore how METTL3 regulates Snail, we first observed that silencing METTL3 led to a decrease in m6A modification levels on SNAIL mRNA using m6A qPCR technology (Fig. 5a). Subsequently, to investigate the impact of m6A modification on SNAIL RNA, we detected the expression levels of SNAIL precursor mRNA (pre-mRNA) and mature mRNA in sh-METTL3-treated cells. The results showed that both pre-mRNA and mature mRNA levels of SNAI1 were significantly higher in the sh-METTL3 group than in the control group (Fig. 5b). Furthermore, subcellular localization analysis of SNAI1 mRNA in sh-METTL3 and sh-NC cells revealed no significant differences between the two groups (Fig. 5c).

To assess the stability of SNAI1 pre-mRNA and mature mRNA, we used Act-D to inhibit new RNA transcription. The experimental results showed that the half-life of pre-mRNA and mature mRNA in the sh-METTL3 group was significantly longer than that in the control group (Fig. 5d). Given that YTHDF2 is known to mediate the degradation of m6A-modified mRNA, we hypothesized that the observed phenomenon might be related to YTHDF2-mediated mRNA degradation. Therefore, we silenced YTHDF2 in colorectal cancer cell lines and observed an increase in the stability of SNAIL mRNA (Fig. 5e).

However, we also found some contradictory phenomena. Despite the increased expression of SNAIL mRNA in the sh-METTL3 group, previous research indicated a decrease in SNAIL protein expression in this group. We speculated that this difference might be due to the influence of m6A on SNAIL mRNA translation efficiency. Therefore, we investigated YTHDF1, which serves as a "reader" of m6A and can recognize m6A-modified mRNA, enhancing its translation [32]. To further explore this mechanism, we conducted experiments with si-YTHDF1 in colorectal cancer cell lines. The results showed that knocking down YTHDF1 significantly reduced SNAIL protein expression (Fig. 5f).

### SNAIL induces CXCL2 expression via the NF-κΒ pathway

**Fig. 6:**
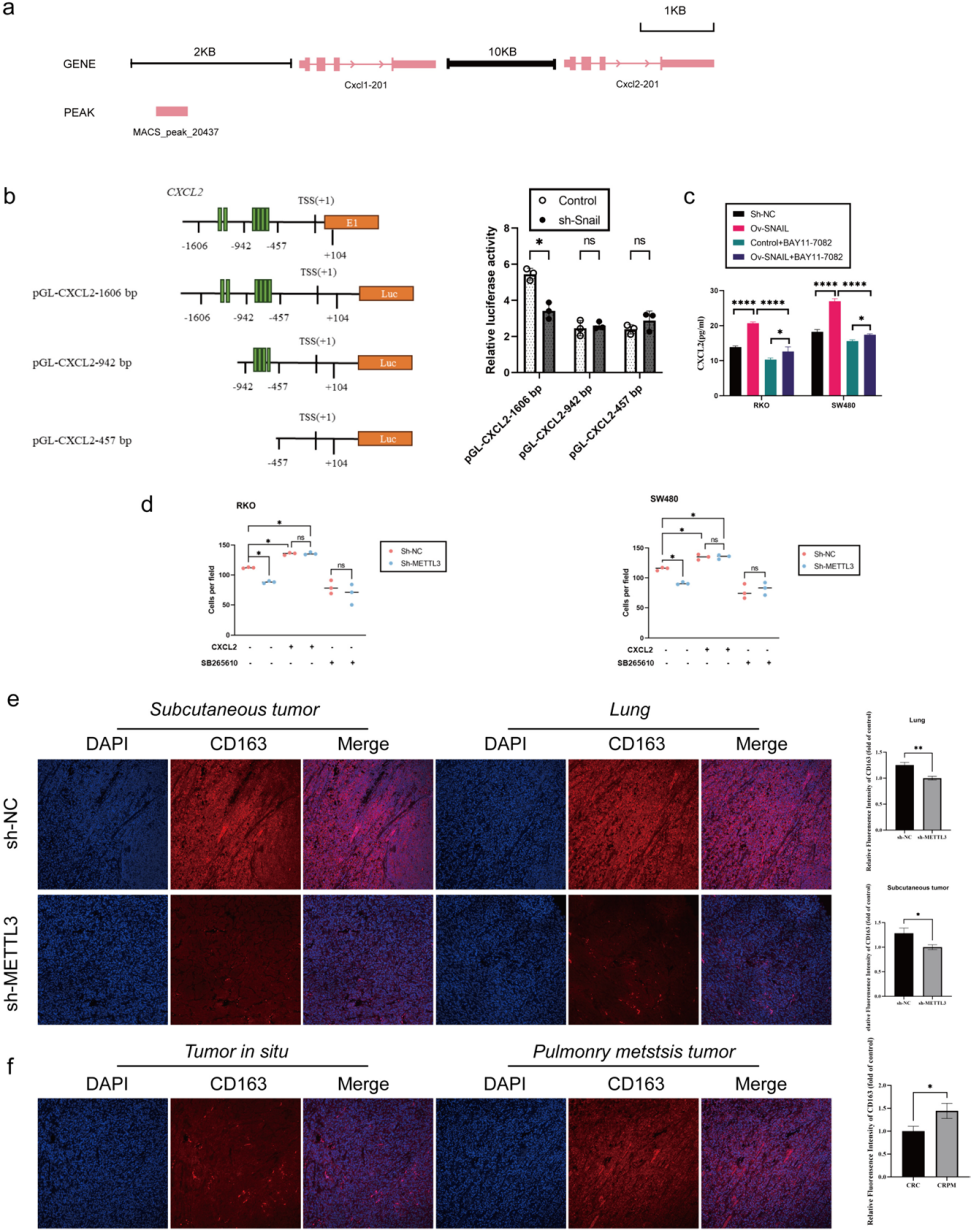
METTL3-expressing CRC Cells Recruits M2-type macrophage Via Secreting CXCL2. **a** Chromatin immunoprecipitation sequence data show that Snail might directly bind to Cxcl2 proximal promoters. **b** (Left) Schematic representation of CXCL2 promoter organization, and the luciferase reporter constructs pGL-CXCL2 (1606 bp: − 1606 to + 104 bp, 942 bp: − 942 to + 104 bp, 457 bp: − 457 to + 104 bp). TSS: transcriptional start site, E1: exon1, and Luc: luciferase. The green bars indicate E-boxes (CANNTG), which are the binding sites of Snail. (Right) Relative luciferase activities are shown. **c** ELISA to analyze the expression of CXCL2 in sh-NC/sh-SNAIL RKO cells (left), and the expression of CXCL2 in sh-NC/sh-SNAIL RKO cells (right), treated with or without BAY11-7082 (NF-κB inhibitor) at 10 μM for 24 h. **d** M2-type macrophage were seeded in the top chamber of the transwell containing 100 mL 1640 medium with or without CXCR2 inhibitor (SB265610, 10 mM). On the other hand, the bottom chamber contained 600 mL of CRC cell conditioned medium (no fetal bovine serum) with or without recombinant CXCL2 protein (1 ng/mL). After 4-h incubation, cells that have completely migrated to the bottom chamber were counted. **e** Immunofluorescence analysis of CD163 protein expression in colorectal in nude mice specimen. **f** Immunofluorescence analysis of CD163 protein expression in colorectal in situ carcinoma and pulmonary metastatic carcinoma. *P < 0.05, **P < 0.01, ***P < 0.001, ****P < 0.0001

Our previous research has revealed a regulatory relationship between SNAIL and CXCL2, but the specific regulatory mechanism remains unclear. In previous sequencing analyses, we noticed significant changes in the NF-κB pathway in the context of lung metastasis cancer (CRPM) compared to colorectal cancer (CRC). Furthermore, through gene set enrichment analysis (GSEA) of CRC data from the TCGA database, we found that in samples with high SNAIL expression, the NF-κB pathway and chemokine signaling pathway were activated, accompanied by epithelial-mesenchymal transition (EMT) (Fig. 5g). Based on these preliminary findings, we proposed a hypothesis: Snail may regulate the expression of CXCL2 through the NF-κB pathway. To validate this hypothesis, we conducted silencing experiments using sh-Snail in SW480 and RKO cell lines and observed a significant decrease in phosphorylated P65 (Phospho-P65) levels (Fig. 5h). Additionally, we treated control group cells with the NF-κB inhibitor BAY11-7082 and found that CXCL2 expression also significantly decreased (Fig. 6c). Furthermore, we analyzed CHIP-seq data (GSE61198) from the Gene Expression Omnibus (GEO) database and found that Snail could bind to the transcription start site (TSS) upstream of CXCL2 (Fig. 6a). To further explore the direct regulatory relationship between SNAIL and CXCL2, we constructed luciferase reporter gene vectors for different lengths of the CXCL2 promoter (pGL-CXCL2-1606 bp, 942 bp, and 457 bp, respectively). The experimental results showed that silencing Snail significantly reduced the activity of the CXCL2-1606 bp promoter, while it had no significant effect on the activity of the CXCL2-942 bp and CXCL2-457 bp promoters (Fig. 6b).

### METTL3-expressing CRC cells recruits M2-type macrophage via secreting CXCL2

Immunohistochemical analysis of subcutaneous tumors and lung metastatic tumors in mice from the sh-METTL3 and sh-NC groups indicated that silencing METTL3 significantly reduced the expression of the M2 macrophage marker CD163 protein (Fig. 6e). Similarly, in clinical samples, the protein expression of the M2 macrophage marker CD163 was significantly higher in lung metastatic tumors compared to primary tumors (Fig. 6f). Next, we evaluated the role of the METTL3-CXCL2 axis in the chemotaxis of M2 macrophages in an in vitro M2 macrophage migration assay. METTL3 knockout significantly reduced the migration of M2 macrophages toward conditioned media derived from RKO and SW480 cells. The addition of recombinant CXCL2 protein rescued M2 macrophage migration in METTL3 knockout cells. On the other hand, blocking the CXCL2 receptor CXCR2 eliminated the difference in mediating M2 macrophage migration between the control and METTL3 knockout conditioned media (Fig. 6d).

## Discussion

METTL3, as a core component of the multifaceted m6A methyltransferase complex (MTC), has been reported to play critical roles in various cancer types [33–38]. While alternative views have been proposed by other studies [39–43], our research findings reveal a significant oncogenic role for METTL3 in the process of tumorigenesis. Previous research has suggested that in certain cancers, METTL3 may function as a tumor suppressor gene. Given the controversies surrounding the roles of m6A modification and METTL3 in different cancer types, our study underscores the potential involvement of METTL3 in colorectal cancer. Our findings demonstrate that METTL3 promotes proliferation, invasion, migration, and inhibits apoptosis in colorectal cancer, highlighting its oncogenic potential. This further emphasizes the widespread impact of METTL3 and m6A methylation in cancer development and precision therapy.

In the field of oncology, epithelial-mesenchymal transition transcription factors (EMT-TFs) such as SNAIL play pivotal roles. SNAIL suppresses the expression of E-cadherin by binding to the e-boxes in the CDH1 promoter and recruiting the multisubunit repressor complex, a crucial process in tumor cells [44, 45]. Aberrant Snail expression is closely associated with EMT, which, in turn, is linked to the invasion, migration, metastasis, settlement, and growth capabilities of tumors, ultimately promoting the formation of CRPM [46]. In the field of colorectal cancer research, prior investigations have already elucidated the regulatory relationship between METTL3 and SNAIL [47]. However, these studies were limited to in vitro research, leaving unanswered the question of whether METTL3 continues to exert a similar influence in the context of colorectal cancer lung metastasis. Consequently, our study is focused on elucidating the m6A modification of SNAIL mRNA by METTL3 and its subsequent ramifications in this specific context. We found that m6A modification enhances the degradation of SNAIL mRNA, which may be related to YTHDF2 specifically recognizing m6A-modified SNAIL mRNA and promoting its degradation [48]. However, this finding contradicts our experimental results, where m6A modification of SNAIL is associated with increased protein expression, raising our concern. Additionally, through sequencing and clinical sample testing, we observed elevated expression levels of METTL3 and YTHDF1 in samples of colorectal cancer lung metastasis. YTHDF1, another m6A "reader," has been found to recognize m6A-modified mRNA and enhance the translation of its targets [32]. To validate the role of YTHDF1, we silenced YTHDF1, and the results showed a significant reduction in SNAIL protein expression, suggesting that m6A methylation regulation of SNAIL is a dynamic process involving multiple factors that require further discussion. Furthermore, our research has also revealed the role of SNAIL in regulating the tumor microenvironment [31]. SNAIL not only recruits M2 macrophages to infiltrate the tumor center by promoting the secretion of CXCL2 but also indirectly regulates the expression of CXCL2 through the NF-κB pathway and directly modulates CXCL2 expression by binding to its promoter region. Moreover, colorectal cancer cells expressing METTL3 recruit M2 macrophages by secreting CXCL2, a finding confirmed through in vitro experiments and in vivo experiments with METTL3-silenced colorectal cancer cells. These findings provide a new perspective for clinical treatment, such as disrupting this malignant cycle using CXCR2 inhibitors.

In summary, our study aimed to elucidate the role and mechanism of RNA m6A methyltransferase METTL3 in CRPM. The results indicate that METTL3 plays a critical role in promoting the development of CRPM by targeting the m6A-Snail-CXCL2 axis, which is involved in recruiting M2 immunosuppressive macrophages. It is worth noting that we also observed a significant reduction in tumor migration upon inhibiting METTL3, suggesting that therapeutic strategies targeting METTL3 may be an effective approach for treating CRPM.

## Data Availability

The analyzed data sets generated during the present study are available from the corresponding author on reasonable request.

## Abbreviations

ALKBH5: AlkB homologue 5
ATCC: American Type Culture Collection
CHIP: Chromatin immunoprecipitation
COAD: Colon adenocarcinoma
CRC: Colorectal cancer
CRPM: Colorectal cancer pulmonary metastasis
ECM: Extracellular matrix
ELISA: Enzyme linked immunosorbent assay
EMT: Epithelial-mesenchymal transition
FTO: Fat mass and obesity-related protein
GEO: Gene Expression Omnibus
HNRNP: Heterogeneous nuclear ribonucleoprotein
IGF2BPs: Insulin-like growth factor 2 mRNA-binding proteins
IHC: Immunohistochemistry
METTL3: Methyltransferase-like 3
MeRIP qRT-PCR: Methylated RNA immunoprecipitation quantitative reverse transcription polymerase chain reaction
mRNA: Messenger RNA
m6A: N6-methyladenosine
PMA: Phorbol 12-myristate 13-acetate
qRT-PCR: Quantitative reverse transcription PCR
SNAIL: Snail family transcriptional repressor 1
TCGA: The Cancer Genome Atlas
TWIST1: Twist family bHLH transcription factor 1
WB: Western blotting
WERs: Writers, erasers, and readers
WTAP: Wilms tumor 1 associated protein
ZEB: Zinc finger E-box-binding homeobox 1

## References

[1] Biller LH, Schrag D (2021). Diagnosis and treatment of metastatic colorectal cancer: a review. JAMA-J AM MED ASSOC.

[2] Zhou H, Liu Z, Wang Y, Wen X, Amador EH, Yuan L, Ran X, Xiong L, Ran Y, Chen W (2022). Colorectal liver metastasis: molecular mechanism and interventional therapy. Signal Transduct Tar.

[3] Chandra R, Karalis JD, Liu C, Murimwa GZ, Voth PJ, Heid CA, Reznik SI, Huang E, Minna JD, Brekken RA. The colorectal cancer tumor microenvironment and its impact on liver and lung metastasis. Cancers. 2021;13(24):6206. 10.3390/cancers13246206 PMC8699466 34944826

[4] Shin AE, Giancotti FG, Rustgi AK (2023). Metastatic colorectal cancer: mechanisms and emerging therapeutics. Trends Pharmacol Sci.

[5] Verstappe J, Berx G (2023). A role for partial epithelial-to-mesenchymal transition in enabling stemness in homeostasis and cancer. Semin Cancer Biol.

[6] Pastushenko I, Blanpain C (2019). EMT Transition States during Tumor Progression and Metastasis. Trends Cell Biol.

[7] Goossens S, Vandamme N, Van Vlierberghe P, Berx G (2017). EMT transcription factors in cancer development re-evaluated: Beyond EMT and MET. BBA-REV Cancer.

[8] Wang Y, Shi J, Chai K, Ying X, Zhou BP (2013). The role of snail in EMT and tumorigenesis. Curr Cancer Drug Tar.

[9] Bakir B, Chiarella AM, Pitarresi JR, Rustgi AK (2020). EMT, MET, plasticity, and tumor metastasis. Trends Cell Biol.

[10] Lu W, Kang Y (2019). Epithelial-mesenchymal plasticity in cancer progression and metastasis. Dev Cell.

[11] Huang Y, Hong W, Wei X (2022). The molecular mechanisms and therapeutic strategies of EMT in tumor progression and metastasis. J Hematol Oncol.

[12] Dongre A, Weinberg RA (2019). New insights into the mechanisms of epithelial-mesenchymal transition and implications for cancer. Nat Rev Mol Cell Bio.

[13] Vu T, Datta PK. Regulation of EMT in colorectal cancer: a culprit in metastasis. Cancers. 2017;9(12):171. 10.3390/cancers9120171 PMC5742819 29258163

[14] Wiener D, Schwartz S (2021). The epitranscriptome beyond m(6)A. Nat Rev Genet.

[15] He L, Li H, Wu A, Peng Y, Shu G, Yin G (2019). Functions of N6-methyladenosine and its role in cancer. Mol Cancer.

[16] He PC, He C (2021). m(6) A RNA methylation: from mechanisms to therapeutic potential. EMBO J.

[17] Wang T, Kong S, Tao M, Ju S (2020). The potential role of RNA N6-methyladenosine in cancer progression. Mol Cancer.

[18] Fang Z, Mei W, Qu C, Lu J, Shang L, Cao F, Li F (2022). Role of m6A writers, erasers and readers in cancer. Exp Hematol Oncol.

[19] Deng LJ, Deng WQ, Fan SR, Chen MF, Qi M, Lyu WY, Qi Q, Tiwari AK, Chen JX, Zhang DM (2022). m6A modification: recent advances, anticancer targeted drug discovery and beyond. Mol Cancer.

[20] Jiang X, Liu B, Nie Z, Duan L, Xiong Q, Jin Z, Yang C, Chen Y (2021). The role of m6A modification in the biological functions and diseases. Signal Transduct Tar.

[21] Zhang Y, Chen W, Zheng X, Guo Y, Cao J, Zhang Y, Wen S, Gao W, Wu Y (2021). Regulatory role and mechanism of m(6)A RNA modification in human metabolic diseases. Mol Ther-Oncolytics.

[22] Oerum S, Meynier V, Catala M, Tisné C (2021). A comprehensive review of m6A/m6Am RNA methyltransferase structures. Nucleic Acids Res.

[23] Zhao Y, Shi Y, Shen H, Xie W (2020). m(6)A-binding proteins: the emerging crucial performers in epigenetics. J Hematol Oncol.

[24] Azzam SK, Alsafar H, Sajini AA. FTO m6A demethylase in obesity and cancer: implications and underlying molecular mechanisms. Int J Mol Sci. 2022;23(7):3800. 10.3390/ijms23073800 PMC8998816 35409166

[25] An Y, Duan H (2022). The role of m6A RNA methylation in cancer metabolism. Mol Cancer.

[26] Sun T, Wu R, Ming L (2019). The role of m6A RNA methylation in cancer. Biomed Pharmacother.

[27] Yue B, Song C, Yang L, Cui R, Cheng X, Zhang Z, Zhao G (2019). METTL3-mediated N6-methyladenosine modification is critical for epithelial-mesenchymal transition and metastasis of gastric cancer. Mol Cancer.

[28] Lin X, Chai G, Wu Y, Li J, Chen F, Liu J, Luo G, Tauler J, Du J, Lin S (2019). RNA m(6)A methylation regulates the epithelial mesenchymal transition of cancer cells and translation of Snail. Nat Commun.

[29] Chen H, Pan Y, Zhou Q, Liang C, Wong CC, Zhou Y, Huang D, Liu W, Zhai J, Gou H (2022). METTL3 inhibits antitumor immunity by targeting m(6)A-BHLHE41-CXCL1/CXCR2 axis to promote colorectal cancer. Gastroenterology.

[30] Li T, Hu PS, Zuo Z, Lin JF, Li X, Wu QN, Chen ZH, Zeng ZL, Wang F, Zheng J (2019). METTL3 facilitates tumor progression via an m(6)A-IGF2BP2-dependent mechanism in colorectal carcinoma. Mol Cancer.

[31] Bao Z, Zeng W, Zhang D, Wang L, Deng X, Lai J, Li J, Gong J, Xiang G (2022). SNAIL induces EMT and lung metastasis of tumours secreting CXCL2 to promote the invasion of M2-type immunosuppressed macrophages in colorectal cancer. Int J Biol Sci.

[32] Wang X, Zhao BS, Roundtree IA, Lu Z, Han D, Ma H, Weng X, Chen K, Shi H, He C (2015). N(6)-methyladenosine modulates messenger RNA translation efficiency. Cell.

[33] Vu LP, Pickering BF, Cheng Y, Zaccara S, Nguyen D, Minuesa G, Chou T, Chow A, Saletore Y, MacKay M (2017). The N(6)-methyladenosine (m(6)A)-forming enzyme METTL3 controls myeloid differentiation of normal hematopoietic and leukemia cells. Nat Med.

[34] Barbieri I, Tzelepis K, Pandolfini L, Shi J, Millán-Zambrano G, Robson SC, Aspris D, Migliori V, Bannister AJ, Han N (2017). Promoter-bound METTL3 maintains myeloid leukaemia by m(6)A-dependent translation control. Nature.

[35] Wang H, Xu B, Shi J (2020). N6-methyladenosine METTL3 promotes the breast cancer progression via targeting Bcl-2. Gene.

[36] Chen M, Wei L, Law CT, Tsang FH, Shen J, Cheng CL, Tsang LH, Ho DW, Chiu DK, Lee JM (2018). RNA N6-methyladenosine methyltransferase-like 3 promotes liver cancer progression through YTHDF2-dependent posttranscriptional silencing of SOCS2. Hepatology.

[37] Zuo X, Chen Z, Gao W, Zhang Y, Wang J, Wang J, Cao M, Cai J, Wu J, Wang X (2020). M6A-mediated upregulation of LINC00958 increases lipogenesis and acts as a nanotherapeutic target in hepatocellular carcinoma. J Hematol Oncol.

[38] Xu H, Wang H, Zhao W, Fu S, Li Y, Ni W, Xin Y, Li W, Yang C, Bai Y (2020). SUMO1 modification of methyltransferase-like 3 promotes tumor progression via regulating Snail mRNA homeostasis in hepatocellular carcinoma. Theranostics.

[39] Cui Q, Shi H, Ye P, Li L, Qu Q, Sun G, Sun G, Lu Z, Huang Y, Yang CG (2017). m(6)A RNA methylation regulates the self-renewal and tumorigenesis of glioblastoma stem cells. Cell Rep.

[40] Liu J, Eckert MA, Harada BT, Liu SM, Lu Z, Yu K, Tienda SM, Chryplewicz A, Zhu AC, Yang Y (2018). m(6)A mRNA methylation regulates AKT activity to promote the proliferation and tumorigenicity of endometrial cancer. Nat Cell Biol.

[41] Jia R, Chai P, Wang S, Sun B, Xu Y, Yang Y, Ge S, Jia R, Yang YG, Fan X (2019). m(6)A modification suppresses ocular melanoma through modulating HINT2 mRNA translation. Mol Cancer.

[42] Deng R, Cheng Y, Ye S, Zhang J, Huang R, Li P, Liu H, Deng Q, Wu X, Lan P (2019). m(6)A methyltransferase METTL3 suppresses colorectal cancer proliferation and migration through p38/ERK pathways. Oncotargets Ther.

[43] Zhao S, Liu J, Nanga P, Liu Y, Cicek AE, Knoblauch N, He C, Stephens M, He X (2019). Detailed modeling of positive selection improves detection of cancer driver genes. Nat Commun.

[44] Batlle E, Sancho E, Francí C, Domínguez D, Monfar M, Baulida J, García DHA (2000). The transcription factor snail is a repressor of E-cadherin gene expression in epithelial tumour cells. Nat Cell Biol.

[45] Cano A, Pérez-Moreno MA, Rodrigo I, Locascio A, Blanco MJ, Del BM, Portillo F, Nieto MA (2000). The transcription factor snail controls epithelial-mesenchymal transitions by repressing E-cadherin expression. Nat Cell Biol.

[46] Brabletz S, Schuhwerk H, Brabletz T, Stemmler MP (2021). Dynamic EMT: a multi-tool for tumor progression. Embo J.

[47] Wen J, Zhang G, Meng Y, Zhang L, Jiang M, Yu Z (2021). RNA m(6)A methyltransferase METTL3 promotes colorectal cancer cell proliferation and invasion by regulating snail expression. Oncol Lett.

[48] Wang X, Lu Z, Gomez A, Hon GC, Yue Y, Han D, Fu Y, Parisien M, Dai Q, Jia G (2014). N6-methyladenosine-dependent regulation of messenger RNA stability. Nature.

